# Whole-genome sequence of African swine fever virus isolated in West Timor, Indonesia

**DOI:** 10.1128/mra.00205-25

**Published:** 2025-08-27

**Authors:** Putri Pandarangga, Maria Gelolodo, Larry Toha, Rick Tearle, Farhid Hemmatzadeh

**Affiliations:** 1 Laboratorium Klinik, Reproduksi, Patologi, dan Nutrisi, Fakultas Kedokteran dan Kedokteran Hewan, Universitas Nusa Cendana 185866, Kupang, Indonesia; 2 Laboratorium Ilmu Penyakit Hewan dan kesehatan Masyarakat, Fakultas Kedokteran dan Kedokteran Hewan, Universitas Nusa Cendana 185866, Kupang, Indonesia; 3 Davies Research Centre, School of Animal and Veterinary Sciences, University of Adelaide 170521, Adelaide, South Australia, Australia; 4 School of Animal and Veterinary Sciences, University of Adelaide 170521, Adelaide, South Australia, Australia; Katholieke Universiteit Leuven, Leuven, Belgium

**Keywords:** ASFV, genotype II, whole-genome sequence, West Timor, Indonesia

## Abstract

African swine fever virus) isolate K1-1/Liver/Kupang/Indonesia/2023 was sequenced from the liver of a dead pig in West Timor, Indonesia. The genome is 186,611 bp long and belongs to genotype II, similar to the isolate from Timor Leste.

## ANNOUNCEMENT

African swine fever (ASF) is a hemorrhagic disease in pigs that causes the death of up to 100% of infected pigs. The disease is caused by the ASF virus (ASFV), belonging to the family *Asfarviridae* and genus *Asfivirus* (1). In 2019, genotype II was responsible for the first ASF outbreaks in Indonesia, which entered through North Sumatra Province (2). At the same time, at the end of 2019, the Western part of Timor Island, which is part of Indonesia, experienced an outbreak killing 23,568 pigs (3). It was speculated that this virus also came from the Eastern part of Timor Island, known as Timor-Leste, only a few weeks after Timor-Leste officially reported the ASF outbreaks (4, 5).

The virus nucleic acids were extracted from the dead pig’s liver in Kupang District, West Timor, Indonesia, using the DNeasy Blood & Tissue Kit (QIAGEN, Germany) with a modified protocol by increasing tissue incubation time in ATL buffer and proteinase K for 1 h while mixing thoroughly every 15 min (6). The library was prepared without PCR amplification, but using fragmented gDNA tagged with an Illumina-compatible adapter using the DNA Library Prep Kit for Illumina (California). The library quality and quantity were determined using a Qubit Fluorometer, Tape Station, and qPCR (The Illumina_Real‐Time PCR System). Sequencing was performed on Illumina NextSeq 500 with 300 cycles (150-PE). The total number of raw reads was 132.2 million. The bioinformatics analysis was modified hereafter from the pipeline designed by Pandarangga et al., with default parameters (7). Any adapters and low-quality reads were filtered and trimmed using Trimmomatic v0.39 (8), and then the read quality was checked with FastQC v0.11.9 (9), resulting in 127.2 million reads. To support the ASFV sequence viral enrichment, Qualimap was used to identify a reference with the highest coverage depth from the NCBI RefSeq database; ASFV isolate Odintsovo_02/14 (accession number NC_044948.1). Then, the filtered reads were mapped to this reference using BWA v0.7.17 (9) and further processed for *de novo* assembly using SPAdes v3.13.1 (10). Contigs less than 1,000 bp in length, with a coverage depth lower than 2, were discarded from the unfiltered scaffolds. Hence, the final genome of the ASFV Kupang strain had a total read length of 186,611 bp; a coverage depth of 60.935-fold; and a 38.4% GC content. Taxonomy annotation was performed using the BLAST web server using the nt/nr databases from NCBI. The result shows 97.81% identity with the genotype II strain from Vietnam (ASFV strain VN/HY-ASFV1 (2019); accession number: MT872723.1). Therefore, the isolate from West Timor, Indonesia (isolate K1-1/Liver/Kupang/Indonesia/2023) also belongs to genotype II. Moreover, the ASFV circulating in Kupang may serve as a good candidate for the development of a homologous vaccine, as was done for the Newcastle Disease vaccine in Indonesia (11, 12).

## Data Availability

The African swine fever virus isolate K1-1/Liver/Kupang/Indonesia/2023 has been submitted to NCBI under BioProject number: PRJNA983205, SRA number: SRR25080344, and accession number: OR604566.1.

## References

[1] Alonso C, Borca M, Dixon L, Revilla Y, Rodriguez F, Escribano JM, Ictv Report Consortium. 2018. ICTV virus taxonomy profile: Asfarviridae. J Gen Virol 99:613–614. doi:10.1099/jgv.0.001049 29565243 PMC12662184

[2] Dharmayanti NI, Sendow I, Ratnawati A, Settypalli TBK, Saepulloh M, Dundon WG, Nuradji H, Naletoski I, Cattoli G, Lamien CE. 2021. African swine fever in North Sumatra and West Java provinces in 2019 and 2020, Indonesia. Transbound Emerg Dis 68:2890–2896. doi:10.1111/tbed.14070 33725423

[3] de Rosary E. 2021. Setahun Lebih Virus ASF Serang Ternak Babi di NTT. Apa yang Harus Dilakukan? Available from: https://www.mongabay.co.id/2021/03/22/setahun-lebih-virus-asf-serang-ternak-babi-di-ntt-apa-yang-harus-dilakukan/

[4] Presidency of the Council of Ministers. 2019. African swine fever in Timor-Leste. Available from: http://timor-leste.gov.tl/?p=23069&lang=en

[5] Mileto P, da Conceição F, Stevens V, Cummins D, Certoma A, Neave MJ, Bendita da Costa Jong J, Williams DT. 2021. Complete genome sequence of African swine fever virus isolated from a domestic pig in Timor-Leste, 2019. Microbiol Resour Announc 10:e0026321. doi:10.1128/MRA.00263-21 34197195 PMC8248885

[6] Pandarangga P, Ticoalu AE, Gelolodo M, Toha LRW. 2023. Development of highly sensitive conventional PCR for African swine fever virus diagnosis in east Nusa Tenggara (NTT) Provinc. J Kaji Vet 11:218–224. doi:10.35508/jkv.v11i2.13118

[7] Pandarangga P, Cahyono MI, McAllister MM, Peaston AE, Tearle R, Low WY, Doan PTK, Rabiei M, Ignjatovic J, Dharmayanti NPI, Indriani R, Tarigan S, Hemmatzadeh F. 2020. Full-genome sequences of two Newcastle disease virus strains isolated in West Java, Indonesia. Microbiol Resour Announc 9:e00221-20. doi:10.1128/MRA.00221-20 32527784 PMC7291095

[8] Bolger AM, Lohse M, Usadel B. 2014. Trimmomatic: a flexible trimmer for Illumina sequence data. Bioinformatics 30:2114–2120. doi:10.1093/bioinformatics/btu170 24695404 PMC4103590

[9] Andrews S. 2010. FastQC: a quality control tool for high throughput sequence data. Babraham Bioinformatics, Babraham Institute, Cambridge, United Kingdom.

[10] Bankevich A, Nurk S, Antipov D, Gurevich AA, Dvorkin M, Kulikov AS, Lesin VM, Nikolenko SI, Pham S, Prjibelski AD, Pyshkin AV, Sirotkin AV, Vyahhi N, Tesler G, Alekseyev MA, Pevzner PA. 2012. SPAdes: a new genome assembly algorithm and its applications to single-cell sequencing. J Comput Biol 19:455–477. doi:10.1089/cmb.2012.0021 22506599 PMC3342519

[11] Pandarangga P, McAllister MM, Peaston AE, Ngai YT, Cahyono MI, Hemmatzadeh F. 2022. Performance comparison of homologous and heterologous Newcastle disease virus in vaccines and antibody tests. Res Vet Sci 149:82–89. doi:10.1016/j.rvsc.2022.06.014 35777283

[12] Pandarangga Putri, Doan PTK, Tearle R, Low WY, Ren Y, Nguyen HTH, Dharmayanti NI, Hemmatzadeh F. 2024. mRNA profiling and transcriptomics analysis of chickens received Newcastle disease virus genotype II and genotype VII vaccines. Pathogens 13:638. doi:10.3390/pathogens13080638 39204239 PMC11357267

